# The potential role of NLRP3 inflammasome in integrating redox, metabolic, and inflammatory signals during follicular development: a hypothesis-driven redox-metabolic integrator framework

**DOI:** 10.3389/fcell.2026.1914362

**Published:** 2026-08-27

**Authors:** Zhenghong Zhang, Defan Wang, Qinghe Lin, Pingting Guo, Zhengchao Wang

**Affiliations:** 1 Provincial Key Laboratory for Developmental Biology and Neurosciences, Key Laboratory of Optoelectronic Science and Technology for Medicine of Ministry of Education, College of Life Sciences, Fujian Normal University, Fuzhou, China; 2 Fujian Provincial Key Laboratory of Reproductive Health Research, School of Medicine, Xiamen University, Xiamen, China; 3 College of Animal Science, Fujian Agriculture and Forestry University, Fuzhou, China

**Keywords:** follicular atresia, follicular development, NLRP3 inflammasome, ovarian ovulation, oxidative stress

## Abstract

Follicular development is a complex and dynamic biological process involving coordinated interactions among endocrine regulation, cellular metabolism, redox homeostasis, mitochondrial function, and immune signaling. Increasing evidence indicates that oxidative stress, metabolic remodeling, and inflammatory responses are closely interconnected within the ovarian microenvironment and collectively influence follicular developmental outcomes. The NLRP3 inflammasome, traditionally recognized as an intracellular sensor of danger-associated signals, has recently emerged as an important mediator linking oxidative stress, mitochondrial dysfunction, metabolic disturbances, and inflammatory activation. Within ovarian tissues, NLRP3 activation has been associated with granulosa cell function, inflammatory remodeling, follicular atresia, ovulatory processes, and reproductive disorders. In this review, we summarize current knowledge regarding the regulation and functional implications of NLRP3 signaling during follicular development, with particular emphasis on its interactions with redox metabolism, mitochondrial homeostasis, and inflammatory pathways. Based on currently available evidence, we propose a hypothesis-driven framework in which NLRP3 may act as a candidate redox-metabolic integrative node that coordinates stress-related signals within the follicular microenvironment and may contribute to developmental transitions including follicular growth, dominance acquisition, ovulatory competence, and atresia. Importantly, this framework does not suggest that NLRP3 independently determines follicular fate. Rather, it provides a conceptual model describing how NLRP3-associated inflammatory signaling may interact with endocrine, metabolic, and redox networks to influence follicular developmental trajectories. The proposed framework remains to be validated through cell-specific genetic models, temporal intervention studies, and quantitative molecular profiling approaches. Understanding the potential integrative role of NLRP3 may provide new insights into the mechanisms underlying ovarian disorders, including polycystic ovary syndrome, primary ovarian insufficiency, and ovarian aging, while highlighting potential directions for future therapeutic exploration.

## 1 Introduction

Follicular development is a highly coordinated and dynamic biological process that underpins female fertility and reproductive longevity. Throughout reproductive life, ovarian follicles undergo a series of tightly regulated developmental transitions, including primordial follicle activation, pre-antral and antral follicle growth, dominant follicle selection, ovulation, and, for the majority of follicles, atresia. The successful execution of these sequential events determines oocyte competence, endocrine homeostasis, reproductive capacity, and ultimately ovarian lifespan (Mumusoglu et al., 2025; Gershon and Dekel, 2020; Tang et al., 2017; Wu et al., 2016; Wu et al., 2015; Zhang et al., 2012). Rather than representing a predetermined developmental program, follicular fate is continuously shaped by the local ovarian microenvironment, in which endocrine signals, metabolic activity, oxidative status, immune responses, and intercellular communication dynamically interact to determine whether an individual follicle continues to grow, undergoes ovulation, or enters irreversible degeneration (Voros et al., 2026a; Voros et al., 2026b; Lin et al., 2026a; Lin et al., 2026b; Zhang et al., 2023; Xu and Wang, 2021; Wang et al., 2017; Wang et al., 2015; Wang et al., 2012).

Among these regulatory mechanisms, increasing attention has been directed toward the intimate interplay between redox homeostasis, metabolic adaptation, and inflammatory signaling. Physiological levels of reactive oxygen species (ROS) are indispensable for follicular development, contributing to granulosa cell proliferation, oocyte maturation, steroidogenesis, cumulus expansion, and ovulatory tissue remodeling (Weng et al., 2023; Zhang et al., 2026; Moustakli et al., 2025; Yan et al., 2024; Pang et al., 2026). Likewise, the metabolic cooperation between granulosa cells and oocytes ensures adequate energy production to sustain rapid follicular growth, while transient inflammatory responses are now recognized as essential components of normal ovulation and luteal formation. However, disruption of this finely balanced microenvironment profoundly alters follicular development. Excessive oxidative stress induces mitochondrial dysfunction, DNA damage, and cellular apoptosis, whereas metabolic disturbances impair energy homeostasis and further amplify inflammatory responses. These processes reinforce one another to establish a vicious cycle that ultimately compromises follicular competence and accelerates follicular atresi (Pang et al., 2026; Berkel, 2024).

Recent studies suggest that the NLRP3 inflammasome may represent an important molecular interface linking these biological processes. The NLRP3 inflammasome consists of the sensor protein NLRP3, the adaptor ASC, and the effector protease caspase-1. Upon activation by oxidative stress, mitochondrial dysfunction, metabolic perturbations, or diverse danger-associated signals, the assembled inflammasome promotes caspase-1 activation, maturation of IL-1β and IL-18, and gasdermin D-mediated pyroptosis, thereby coordinating inflammatory responses to cellular stress (Moustakli et al., 2025; Berkel, 2024). In the ovary, NLRP3 is expressed in oocytes, granulosa cells, theca cells, and ovarian immune cells, where accumulating evidence implicates its involvement in follicular development, ovulation, ovarian aging, and several reproductive disorders, including polycystic ovary syndrome (PCOS), primary ovarian insufficiency (POI), and endometriosis-associated infertility (Weng et al., 2023; Zhang et al., 2026; Moustakli et al., 2025; Yan et al., 2024; Pang et al., 2026; Berkel, 2024; Kavarthapu et al., 2026; Zhang et al., 2019).

Despite these important advances, current knowledge remains largely fragmented. Most previous studies have investigated oxidative stress, metabolic remodeling, inflammatory signaling, or NLRP3 activation as relatively independent biological events. Although numerous reports have demonstrated associations between NLRP3 activation and specific ovarian phenotypes, there is still no unified conceptual framework explaining how diverse redox, metabolic, and inflammatory signals are integrated to influence follicular developmental decisions across different physiological stages. Moreover, the mechanisms through which follicles interpret complex microenvironmental information to determine whether they continue development, acquire ovulatory competence, or undergo atresia remain incompletely understood. Consequently, an integrative systems-level model capable of connecting these interconnected regulatory processes is currently lacking.

To address this conceptual gap, we propose a hypothesis-driven framework in which NLRP3-associated signaling may represent one component of the regulatory network influencing follicular developmental transitions. Rather than viewing NLRP3 solely as an executor of inflammatory responses, this framework considers the inflammasome as a dynamic signaling hub that integrates oxidative stress, metabolic adaptation, mitochondrial status, and innate immune activation to coordinate follicular developmental transitions. We emphasize that this gatekeeper model is intended as a conceptual interpretation of the currently available evidence rather than a definitive mechanistic conclusion. It is designed to provide an integrated perspective for understanding how multiple environmental cues may converge to influence follicular survival, dominant follicle selection, ovulation, and atresia.

In this review, we systematically summarize current knowledge regarding the structure and activation mechanisms of the NLRP3 inflammasome, its roles in redox regulation, metabolic remodeling, and follicular development, as well as its involvement in major ovarian disorders. Building upon these findings, we propose NLRP3 inflammasome as a candidate redox-metabolic integrator during follicular development to illustrate how redox and metabolic signals may be coordinated within the follicular microenvironment. Finally, we discuss the limitations of the current evidence, outline future experimental strategies for validating this conceptual framework, and highlight its potential implications for precision therapeutic interventions targeting female reproductive disorders.

## 2 The structure and activation of NLRP3 inflammasome

The NLRP3 inflammasome is a multiprotein complex composed of the sensor protein NLRP3, the adaptor protein ASC, and the effector protease caspase-1, which assembles in response to cellular stress signals to initiate inflammasome activation (Weng et al., 2023; Zhang et al., 2026).

In the canonical activation pathway, a priming signal like LPS, induces NLRP3 and pro-IL-1β transcriptional upregulation. Subsequently, an activation signal like extracellular ATP, induces NLRP3 conformational rearrangement and oligomerization. Diverse pathogen- or danger-associated molecular patterns can also contribute indirectly to this process. This promotes ASC recruitment and assembly of the inflammasome complex, leading to caspase-1 activation and subsequent cleavage of pro-IL-1β and pro-IL-18 into their mature forms, initiating inflammatory responses (Berkel, 2024).

In the non-canonical activation pathway, intracellular LPS activates caspase-4/5 or caspase-11, which cleave gasdermin D, a pore-forming protein that induces membrane permeabilization and pyroptotic cell lysis. This process results in the release of intracellular danger signals that trigger NLRP3 inflammasome activation, amplifying inflammatory cascades (Pang et al., 2026).

## 3 The redox-metabolic landscape during follicular development

Follicular development relies on metabolic synergy and redox balance between granulosa cells and oocytes. These processes shape metabolic landscape and ROS levels of follicular microenvironment, determining follicular fate (Weng et al., 2023; Zhang et al., 2026; Moustakli et al., 2025; Yan et al., 2024).

### 3.1 Metabolic characteristics of growing follicles

In growing follicles, oocytes exhibit limited intrinsic glycolytic capacity. Consequently, they depend predominantly on granulosa cells for energy provision, establishing a metabolic coupling pattern characterized by granulosa cell glycolysis coupled with oocyte oxidative phosphorylation (Moustakli et al., 2025). Granulosa cells metabolize glucose to generate pyruvate and lactate, which are transferred to the oocyte via gap junctions. The oocyte utilizes these substrates to fuel TCA and oxidative phosphorylation, generating ATP to support meiosis and macromolecular biosynthesis (Lin HA. et al., 2026; Lin et al., 2021). Additionally, fatty acid β-oxidation serves as an auxiliary energy source during follicular development (Moustakli et al., 2025; Ouyang et al., 2026).

### 3.2 Physiological ROS during follicular development

Within follicular microenvironment, physiological ROS are critical signaling mediators regulating follicular development. Upon FSH stimulation, mitochondria and NOX generate physiological ROS, which in turn activate ROS-dependent MAPK/ERK signaling, enhance FSH receptor signaling responsiveness, and promote granulosa cell proliferation and estrogen biosynthesis (Pang et al., 2026; Berkel, 2024). Prior to LH surge, estrogen induces NOX4-mediated ROS production, which regulates cumulus expansion, ovulation-related gene expression, and granulosa cell luteinization. Pharmacological scavenging or inhibition of ROS signaling markedly impairs ovulatory processes (Shkolnik et al., 2011).

Furthermore, mitochondria are key organelles governing energy metabolism and ROS production in follicular cells. They rely on intrinsic quality control mechanisms to preserve mitochondrial and cellular homeostasis during follicular development (Wang et al., 2024; Tian et al., 2022).

## 4 Integration of oxidative stress, metabolism and inflammatory signaling

Accumulating evidence indicates that oxidative stress, cellular metabolism, and inflammatory signaling do not function as independent regulatory modules during follicular development. Instead, they constitute an integrated and highly interconnected biological network that may coordinately regulate follicular fate and function (Agarwal et al., 2005; Dumesic et al., 2015; Richards et al., 2002).

### 4.1 Physiological coupling of redox homeostasis, metabolism, and inflammation during follicular development

Normal follicular development depends on the coordinated integration of three regulatory systems: redox homeostasis, cellular metabolism, and inflammatory signaling. Rather than functioning independently, these systems constitute an integrated regulatory network that preserves follicular homeostasis by coordinating energy production, redox balance, and tissue remodeling throughout follicular development (Agarwal et al., 2005; Ruder et al., 2008).

Physiological ROS levels are not simply by-products of cellular metabolism but function as essential signaling molecules during follicular development. Under physiological conditions, low concentrations of ROS are generated primarily through mitochondrial oxidative phosphorylation and NADPH oxidase activity and are tightly controlled by antioxidant defense systems, including SOD, GPX and CAT. Physiological ROS modulate FSH- and LH-mediated signaling, promote granulosa cell proliferation, steroidogenesis, and oocyte maturation, and function as indispensable second messengers for normal follicular development (Shkolnik et al., 2011; Hanukoglu, 2006).

Physiological metabolism provides the energetic and biosynthetic flexibility required to sustain follicular development. Granulosa cells predominantly rely on glycolysis to generate lactate and pyruvate, which are transferred to the oocyte as metabolic substrates. In contrast, oocytes primarily depend on mitochondrial oxidative phosphorylation to generate ATP, thereby meeting the substantial energetic demands of meiotic maturation and subsequent fertilization. This metabolic cooperation ensures efficient energy production while limiting excessive ROS generation, thereby preserving coordination between cellular metabolism and redox homeostasis (Sugiura and Eppig, 2005; Su et al., 2012).

A tightly regulated physiological inflammatory response also represents an essential component of follicular development. Accumulating evidence indicates that ovulation is fundamentally a tightly controlled, localized inflammatory-like process (Espey, 1994). Following LH surge, follicles transiently produce inflammatory mediators including IL-1β, IL-6, TNF-α, prostaglandins, and chemokines, which promote immune cell recruitment, extracellular matrix remodeling, increased vascular permeability, and follicular rupture, thereby facilitating ovulation and subsequent corpus luteum formation (Richards, 2005). Unlike pathological inflammation, this response is spatially and temporally restricted and resolves rapidly after ovulation, thereby preventing excessive tissue injury.

Collectively, physiological ROS provide signaling cues, cellular metabolism supplies the energy and biosynthetic resources required for follicular growth, and transient inflammatory signaling orchestrates tissue remodeling. The coordinated interaction among these three regulatory systems maintains follicular homeostasis and supports normal follicular development.

### 4.2 Oxidative stress and metabolic dysfunction establish a feed-forward pathogenic loop

Under prolonged pathological conditions including aging, metabolic disorders, environmental toxicants, and chronic inflammation, redox homeostasis is among the earliest regulatory systems to become disrupted. Persistent accumulation of ROS beyond the cellular antioxidant capacity not only causes oxidative damage to DNA, lipids and proteins, but also compromises mitochondrial function by reducing electron transport chain efficiency and impairing oxidative phosphorylation (Murphy, 2009).

Mitochondrial dysfunction subsequently reduces ATP production and disrupts TCA flux, resulting in intracellular succinate accumulation. To compensate for impaired oxidative phosphorylation, cells increase glycolytic flux, resulting in enhanced lactate production and progressive metabolic reprogramming toward glycolytic metabolism (Martínez-Re et al., 2020; Brooks, 2020). Rather than restoring metabolic homeostasis, this adaptive response further compromises mitochondrial efficiency, promotes electron leakage, and increases mitochondrial ROS production, thereby amplifying oxidative stress. Consequently, a self-reinforcing positive feedback loop is established between oxidative stress and metabolic dysfunction.

This feed-forward cycle establishes a mutually reinforcing pathological network linking oxidative stress and metabolic dysfunction. ROS exacerbate metabolic dysfunction, whereas metabolic dysfunction generates additional ROS, driving the transition from reversible cellular stress to persistent pathological remodeling. At this stage, inflammatory signaling has not yet become the dominant pathological driver. Instead, disease progression is primarily sustained by the bidirectional amplification between oxidative stress and metabolic dysfunction.

### 4.3 NLRP3 integrates redox and metabolic signals into inflammatory activation

Although oxidative stress and metabolic dysfunction account for the early events leading to follicular impairment, a molecular signaling platform is required to integrate these diverse stress signals and convert them into a coordinated inflammatory response. Accumulating evidence identifies NLRP3 inflammasome as this critical immunometabolic integration platform (Swanson et al., 2019; Kelley et al., 2019).

Unlike classical pattern-recognition receptors that recognize specific ligands, NLRP3 inflammasome senses a wide range of intracellular danger signals generated by metabolic and cellular stress. Representative activating signals include ROS-mediated dissociation of TXNIP from TRX complex, enabling TXNIP to bind NLRP3 and initiate inflammasome assembly; extracellular ATP-induced K+ efflux through the P2X7 receptor, which provides a conserved ionic trigger for activation; mitochondrial release of oxidized mtDNA, functioning as a DAMP; succinate accumulation, which stabilizes HIF-1α and enhances transcription of IL-1β and NLRP3-related genes; and lysosomal disruption, which releases cathepsin B and provides an additional danger signal that collectively promotes inflammasome activation (Zhou et al., 2010; Muñoz-Planillo et al., 2013; Shimada et al., 2012; Tannahill et al., 2013; Hornung et al., 2008; Wang et al., 2025a; Wang et al., 2025b).

Thus, the principal biological role of NLRP3 inflammasome extends beyond functioning as a downstream inflammatory effector; it may function as a central signal integrator that converges diverse metabolic and oxidative danger signals into a unified inflammatory program. Accordingly, NLRP3 inflammasome functions as the molecular hub linking oxidative stress and metabolic dysfunction to inflammatory activation.

### 4.4 Feed-forward amplification between inflammation, oxidative stress, and metabolic remodeling

NLRP3-mediated inflammatory activation does not represent the terminal event of this pathogenic process. Instead, the release of inflammatory mediators further remodels cellular redox homeostasis and metabolic networks, amplifying inflammatory signaling and establishing a self-sustaining feed-forward pathogenic circuit.

Following its maturation and release, IL-1β activates the IL-1R/NF-κB signaling pathway, promoting NOX expression and activity and driving additional ROS production. Simultaneously, IL-18 promotes immune cell recruitment and activation while stimulating the production of secondary inflammatory mediators, including TNF-α, IFN-γ, and IL-6. These cytokines further enhance glycolytic metabolism, exacerbate mitochondrial dysfunction, and promote metabolic reprogramming, resulting in sustained succinate accumulation, HIF-1α stabilization, and additional ROS generation (Dinarello, 2011; O'Neill et al., 2016; Vo et al., 2021; Mills et al., 2016; Chandel, 2015; Picard and Shirihai, 2022).

Consequently, inflammatory signaling continuously reinforces oxidative stress and metabolic dysfunction, generating a progressively self-amplifying pathogenic network. This feed-forward circuit highlights the central role of NLRP3 inflammasome not only as a molecular bridge linking oxidative stress and metabolic dysfunction to inflammation but also as a key driver that transforms transient metabolic stress into persistent chronic inflammation.

## 5 Functional role of NLRP3 during follicular development

NLRP3 may serve as a central signaling network that appears to influence follicular fate, although existing evidence mainly supports a regulatory association between NLRP3 activation and follicular outcomes, while direct causal evidence remains limited.

### 5.1 NLRP3 May contribute to follicular survival

Basal NLRP3 activity contributes to the maintenance of ovarian tissue and follicular homeostasis, without triggering excessive inflammatory injury (Moustakli et al., 2025). Moderate NLRP3 activation promotes the processing and release of IL-1β and IL-18, which may not only mediate controlled inflammatory signaling but also participate in tissue remodeling and functional adaptation within ovarian microenvironment (Berkel, 2024).

NLRP3 inhibition or knockdown studies have suggested that altered NLRP3-associated inflammatory signaling may influence granulosa cell responses under pathological conditions (Yan et al., 2024). However, whether NLRP3 directly controls follicular developmental decisions remains unclear.

### 5.2 Potential role of NLRP3 in dominant follicle selection

Dominant follicle selection represents a complex process involving gonadotropin responsiveness, steroidogenic capacity, metabolic adaptation, and survival signaling. Although inflammatory and metabolic alterations associated with NLRP3 activation may occur during follicular development (Pang et al., 2026; Lin HA. et al., 2026; Lin et al., 2021; Zhang et al., 2025), direct evidence demonstrating that NLRP3 determines dominant follicle selection is currently lacking.

Future studies using lineage-specific NLRP3 manipulation combined with longitudinal follicular tracking will be required to determine whether NLRP3 contributes causally to follicular competitiveness or represents a downstream response to follicular selection processes.

### 5.3 NLRP3-mediated follicular atresia

Aberrant NLRP3 activation may function as a key initiating factor for follicular atresia when follicular redox imbalance and metabolic disturbances intensify (Kavarthapu et al., 2026).

Hyperactivated NLRP3 induces granulosa cell death via apoptotic pathways and compromises cell membrane integrity through pyroptosis, leading to the release of pro-inflammatory factors and the initiation of an inflammatory cascade (Moustakli et al., 2025; Ouyang et al., 2026).

In pathological conditions such as POI and PCOS, upregulated NLRP3-caspase-1 signaling within atretic follicles accelerates follicular atresia, suggesting aberrant NLRP3 activation may be a central driver (Weng et al., 2023; Zhang et al., 2026).

### 5.4 NLRP3 in ovulatory rupture

Ovulation is a physiological, tightly regulated inflammatory-like process, where NLRP3 inflammasome contributes to the establishment of the ovulatory threshold and regulates the coordinated rupture of the follicular wall (Chen et al., 2024).

Following LH surge, NLRP3 activation enhances IL-1β secretion and upregulates MMP expression, promoting extracellular matrix remodeling and facilitating follicular rupture and oocyte release (Moustakli et al., 2025; Zaniker et al., 2023; Liu et al., 2021).

Given that LUF syndrome involves impaired follicular rupture despite luteinization, inflammatory dysregulation may represent one potential contributing mechanism. However, direct evidence linking NLRP3 activation to LUF syndrome is currently lacking.

## 6 Hierarchical and reciprocal integration of NLRP3-Associated redox-metabolic signaling networks

**FIGURE 1:**
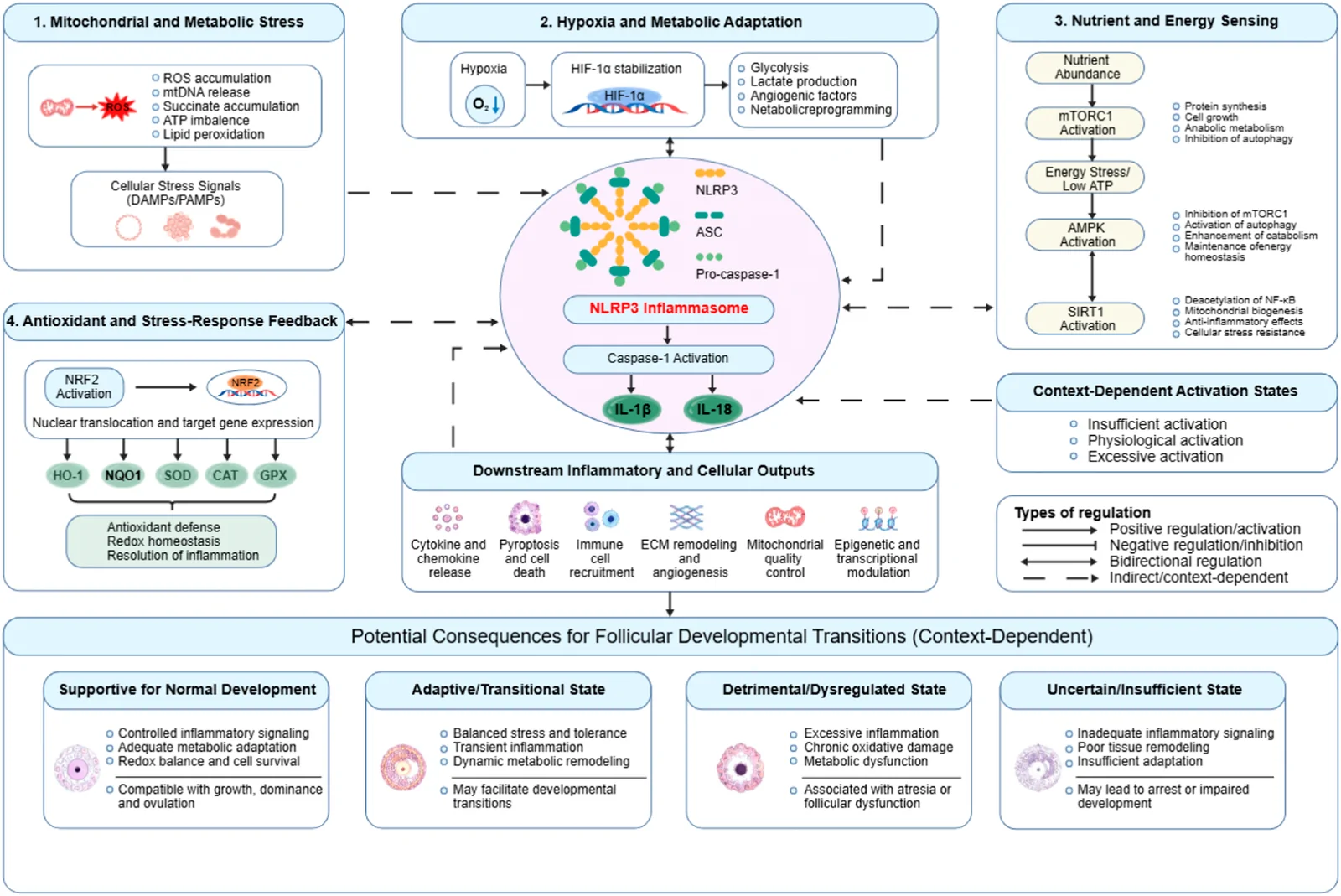
Integration of NLRP3-associated signaling in follicular redox-metabolic-inflammatory network. NLRP3 is proposed as a candidate immunometabolic integrative node that links mitochondrial stress, metabolic remodeling, hypoxic adaptation, nutrient sensing, and antioxidant responses. ROS accumulation, mtDNA release, metabolic alterations, HIF-1α signaling, mTOR/AMPK/SIRT1 pathways, and NRF2-mediated antioxidant feedback may converge on or interact with NLRP3-associated inflammatory signaling in a context-dependent manner. NLRP3 activation may lead to caspase-1 activation and IL-1β/IL-18 maturation, influencing cytokine release, immune communication, extracellular matrix remodeling, and mitochondrial homeostasis. This framework does not propose NLRP3 as an independent determinant of follicular fate but rather as a potential component of a multi-dimensional regulatory network underlying follicular developmental transitions. Further validation using genetic, temporal, and multi-omics approaches is required. Note: This network represents a hypothesis-driven framework. NLRP3 is proposed as a candidate integrative node within a complex signaling network, rather than a master regulator that alone determines follicular fate. The exact contribution of each pathway remains to be experimentally validated.

The regulation of follicular homeostasis cannot be explained by a single linear signaling cascade. Instead, NLRP3-associated inflammatory signaling is embedded within a broader redox-metabolic regulatory network involving HIF-1α, mTOR, NRF2, and AMPK/SIRT1 pathways. These pathways function at different regulatory layers: HIF-1α and AMPK/SIRT1 primarily sense metabolic stress and energy availability, mTOR integrates nutrient and growth signals, NRF2 maintains antioxidant defense, whereas NLRP3 responds to cellular danger signals and contributes to inflammatory amplification. Importantly, these pathways also exert NLRP3-independent effects, indicating that NLRP3 represents one component rather than the central regulator of this network (Figure 1).

### 6.1 Hypoxia inducible factor (HIF)-1α signaling

The developing follicular microenvironment is characterized by relative hypoxia, which promotes HIF-1α stabilization and nuclear accumulation, initiating transcriptional programs (Yan et al., 2024).

By upregulating glycolytic enzymes, HIF-1α promotes glycolytic reprogramming, leading to increased production of lactate and succinate, which facilitate NLRP3 activation (Lin HA. et al., 2026; Lin et al., 2021).

Conversely, NLRP3 activation and IL-1β signaling further enhance HIF-1α expression, establishing a reciprocal NLRP3-HIF-1α positive feedback loop that amplifies metabolic dysregulation and inflammatory signaling.

HIF-1α may interact with NLRP3-associated inflammatory signaling under conditions of metabolic stress. However, HIF-1α also exerts extensive NLRP3-independent effects on metabolic adaptation and tissue homeostasis.

### 6.2 Mammalian target of rapamycin (mTOR) signaling

mTOR is a central nutrient-sensing and growth-regulatory signaling that exhibits functional crosstalk with NLRP3 inflammasome (Zhang et al., 2025).

Under nutrient sufficiency, mTOR is activated and suppresses autophagic flux, impairing the timely clearance of damaged mitochondria and inflammasome-related cellular components. mTOR signaling also enhances NLRP3 expression and facilitates inflammasome activation, amplifying inflammatory responses (Moustakli et al., 2025; Ouyang et al., 2026).

mTOR signaling should be considered an upstream nutrient-sensing pathway that may indirectly modulate inflammatory responses, including NLRP3 activation, while maintaining substantial NLRP3-independent roles in follicular growth and metabolism.

### 6.3 NRF2-mediated antioxidant signaling

NRF2 forms a negative regulatory feedback with NLRP3 signaling to constrain excessive inflammatory activation (Moustakli et al., 2025).

NLRP3 activation increases ROS production, which promotes NRF2 activation and induces SOD and GPX4 expressions. This antioxidant response restores redox homeostasis and suppresses ROS-driven NLRP3 activation, providing a protective mechanism against oxidative damage in follicles (Pang et al., 2026).

NRF2 functions as a major antioxidant and cytoprotective pathway that can influence NLRP3 activation by controlling cellular redox status, but its protective effects extend beyond inflammasome regulation.

### 6.4 AMPK/SIRT1 metabolic signaling

SIRT1 and AMPK form a coordinated cellular energy-sensing network that negatively regulates NLRP3 activity through the maintenance of metabolic homeostasis and mitochondrial integrity (Berkel, 2024).

Under follicular metabolic stress, AMPK and SIRT1 act synergistically to enhance mitochondrial function and reduce ROS production. Concurrently, they suppress NLRP3 and caspase-1 activation, inhibiting the maturation and release of downstream pro-inflammatory cytokines (Kavarthapu et al., 2026).

AMPK/SIRT1 signaling may restrain excessive NLRP3 activation through metabolic and mitochondrial mechanisms, while independently maintaining cellular energy homeostasis.

Together, NLRP3 integrates signals derived from these pathways and translates them into inflammatory responses that ultimately influence follicular survival, growth, ovulation, or atresia. These signaling pathways should not be interpreted as parallel or independent regulators but rather as components of a hierarchical regulatory network centered on NLRP3. Importantly, these interactions are bidirectional rather than linear (Figure 1).

## 7 NLRP3 dysregulation in ovarian disorders

NLRP3 inflammasome is implicated in the pathophysiology of multiple ovarian disorders, including PCOS, POI, and ovarian aging (Weng et al., 2023; Zhang et al., 2026; Berkel, 2024).

### 7.1 Polycystic ovary syndrome (PCOS)

PCOS is a multifactorial endocrine-metabolic disorder characterized by hyperandrogenism, metabolic dysfunction, and abnormal follicular development (Moustakli et al., 2025; Ouyang et al., 2026).

Emerging evidence suggests that inflammatory activation, including NLRP3-associated signaling, may contribute to the ovarian microenvironment associated with PCOS (Weng et al., 2023; Zhang et al., 2026). However, whether NLRP3 represents a causal driver, a disease amplifier, or a secondary response remains unresolved.

The contribution of NLRP3 may differ among PCOS subtypes depending on metabolic status, inflammatory burden, and hormonal environment.

### 7.2 Primary ovarian insufficiency (POI)

In POI-affected ovarian tissue, NLRP3 inflammasome is aberrantly activated. Oxidative stress-induced genomic and mitochondrial DNA damage can promote NLRP3 activation, and the resulting release of pro-inflammatory cytokines further exacerbates mitochondrial dysfunction and impairs DNA repair mechanism. Berkel et al. Demonstrated IL-1β and IL-18 are elevated in follicular fluid of POI patients and correlate with NLRP3 expression and ovarian dysfunction (Berkel, 2024).

Clinical studies have reported alterations in inflammatory mediators and follicular microenvironmental changes in patients with ovarian insufficiency (Berkel, 2024). These findings suggest that inflammatory dysregulation, potentially involving NLRP3-associated pathways, may be associated with impaired ovarian function. However, direct evidence linking follicular fluid NLRP3 activation to POI pathogenesis remains limited.

### 7.3 Ovarian aging

Inflammaging is a core hallmark of ovarian aging, while NLRP3 inflammasome is a central mediator of age-associated inflammatory remodeling. In aging ovaries, ROS accumulation, mtDNA damage and dysfunctional mitochondria contribute to sustained NLRP3 activation, driving chronic inflammatory responses. This state further exacerbates oxidative stress and mitochondrial dysfunction, accelerating follicular atresia and ovarian fibrosis, establishing a feed-forward cycle (Zhou et al., 2024; Grzeczka et al., 2025).

Zhou et al. have demonstrated that NLRP3 expression and activity are elevated in the ovaries of aged mice, while inhibiting NLRP3 reduces follicular apoptosis and ameliorates ovarian dysfunction, supporting the important role of NLRP3 in ovarian aging (Zhou et al., 2024; Grzeczka et al., 2025). Evidence supporting the involvement of NLRP3 in ovarian aging currently derives predominantly from animal studies, whereas clinical validation remains insufficient.

NLRP3-associated inflammatory signaling may represent one component of disease-associated networks in ovarian disorders, rather than a universal causal mechanism.

## 8 A hypothesis-driven framework: potential integration of NLRP3 signaling during follicular developmental transitions

**FIGURE 2:**
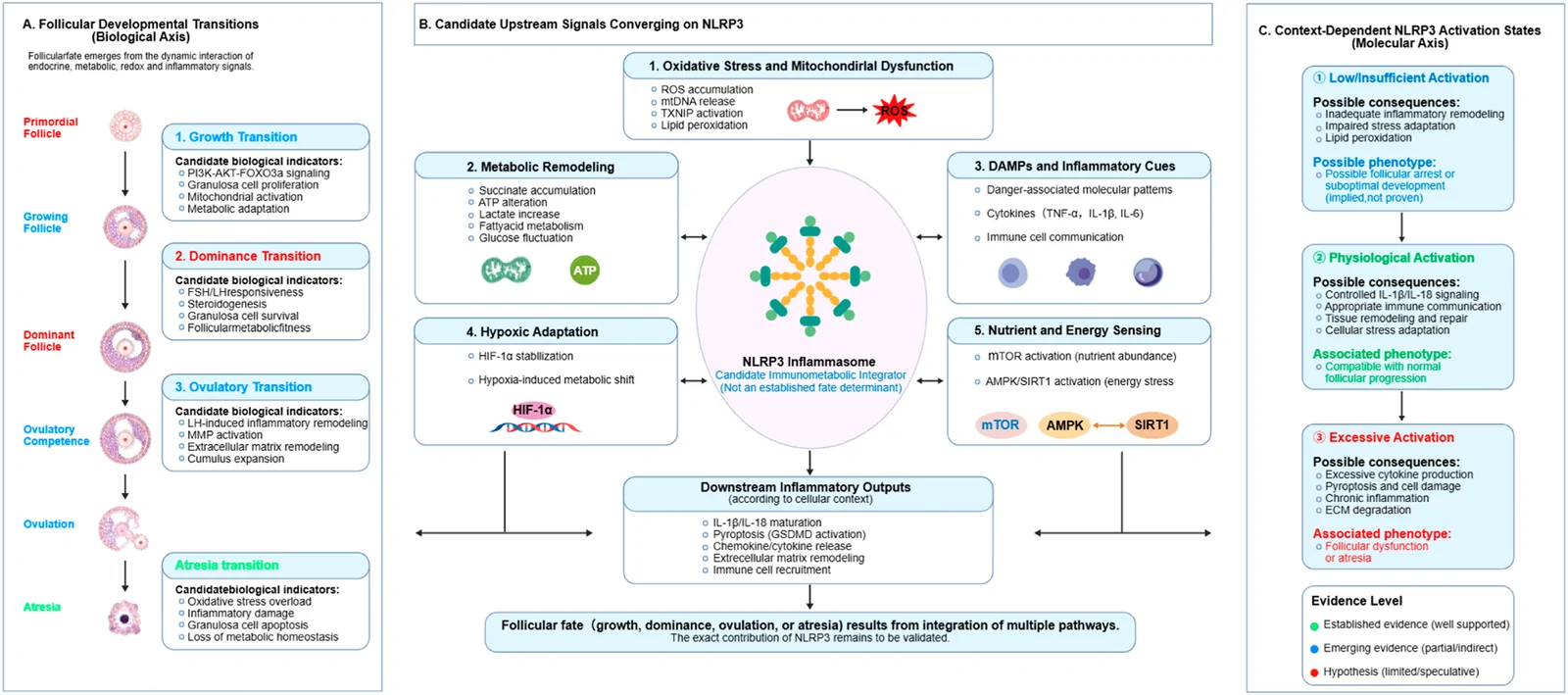
A hypothesis-driven integrative framework illustrating the potential role of NLRP3 as a redox-metabolic signaling node during follicular developmental transitions. Follicular fate is proposed to emerge from the dynamic interaction among endocrine signals, metabolic status, redox homeostasis, mitochondrial function, and inflammatory signaling. In this conceptual framework, follicular development proceeds through several major biological transitions, including primordial follicle activation, follicular growth, acquisition of dominance, ovulatory competence, or atresia. Rather than directly determining follicular fate, NLRP3 inflammasome is proposed as a potential integrative signaling node that senses intracellular stress-associated signals, including reactive oxygen species (ROS), mitochondrial damage-associated molecular patterns (mtDNA), metabolic intermediates, and inflammatory cues. Different cellular contexts and activation states of NLRP3 may influence downstream inflammatory outputs, including IL-1β/IL-18 maturation, pyroptotic responses, extracellular matrix remodeling, and immune communication. The relationship between NLRP3 activity and follicular developmental transitions remains hypothetical and requires validation through cell-specific genetic models, temporal manipulation, and quantitative biomarker analysis. Created with BioGDP.com. Note: This frame work proposes that NLRP3 may act as a redox-metabolic integrative node inffuencing follicular development altransitions. It does not assumet hat NLRP3 directly determines follicular fate. Causality and mechanistic contributions require rigorous experimental validation.

Integrating current experimental observations, we propose a hypothesis-driven framework describing the potential involvement of NLRP3 signaling in follicular developmental transitions. This framework does not define NLRP3 as an independent determinant of follicular fate, but rather considers NLRP3 as a candidate immunometabolic integrator that may coordinate stress-associated signals within the follicular microenvironment (Figure 2).

### 8.1 Concept of developmental transition thresholds

Follicular development is influenced by multiple interacting systems, including endocrine cues, metabolic availability, mitochondrial function, redox balance, and inflammatory remodeling. Within this complex network, NLRP3 activation may represent one component that reflects and responds to changes in cellular states. Therefore, the proposed framework aims to describe how NLRP3-associated signaling could participate in the transition between different developmental states rather than establish a direct causal hierarchy. Rather than representing fixed molecular checkpoints controlled by NLRP3, the proposed thresholds are conceptual transition states reflecting coordinated changes across multiple regulatory systems, including growth transition, dominance transition, and ovulatory transition (Moustakli et al., 2025; Lin HA. et al., 2026; Lin et al., 2021).

The growth transition represents the shift from primordial follicle dormancy toward active follicular development. This process is influenced by nutrient availability, PI3K-AKT-FOXO3a signaling, mitochondrial activity, and granulosa cell metabolic adaptation. NLRP3-associated inflammatory signaling may participate in this process by modulating cellular stress responses; however, whether NLRP3 is required for follicle activation remains experimentally unresolved.

The acquisition of dominant follicle characteristics requires integration of gonadotropin responsiveness, steroidogenic capacity, metabolic fitness, and survival signaling. Although NLRP3 activation has been associated with ovarian inflammatory responses, direct evidence demonstrating that NLRP3 controls dominant follicle selection is currently lacking. Future temporal and cell-specific studies are necessary to determine whether NLRP3 contributes to, reflects, or responds to dominance-associated changes.

Ovulatory competence represents a highly coordinated inflammatory-like remodeling process involving LH signaling, extracellular matrix degradation, cytokine production, and immune cell recruitment. NLRP3-related IL-1β signaling may contribute to inflammatory remodeling during ovulation; however, its necessity and specificity as an ovulatory regulator require further experimental validation.

### 8.2 NLRP3 as a redox-metabolic sensor

NLRP3 inflammasome is a redox-metabolic sensor within follicular microenvironment, forming a dynamic redox-metabolic gate (Yan et al., 2024).

At the redox level, NLRP3 senses oxidative stress-associated signals, including ROS dynamics and mtDNA release. At the metabolic level, it responds to alterations in energy status and lipid-derived metabolites, including ATP, lactate, succinate and FFA (Zhang et al., 2025). Furthermore, NLRP3 interacts with HIF-1α to integrate hypoxia-induced metabolic reprogramming within the follicular niche (Pang et al., 2026).

Upon the diverse signals, NLRP3 transduces these inputs into inflammatory and metabolic outputs that regulate follicular development, serving as a central signaling hub linking microenvironmental dynamics to follicular fate.

### 8.3 Context-dependent NLRP3 signaling states

NLRP3 activation should be considered a context-dependent signaling spectrum rather than a simple molecular switch controlling follicular fate. Insufficient NLRP3-associated signaling may be associated with reduced inflammatory adaptation and impaired tissue remodeling, but direct evidence remains limited. Physiological NLRP3-associated signaling may support controlled cytokine production, immune communication, and tissue remodeling. Excessive NLRP3 activation may contribute to pyroptosis, chronic inflammation, and follicular dysfunction. Chronic dysregulation accelerates ovarian inflammaging and contributes to the pathogenesis of disorders such as PCOS and POI (Zhou et al., 2024; Grzeczka et al., 2025).

This hypothesis-driven framework proposes that NLRP3 may represent an important immunometabolic integration node within the follicular microenvironment. Rather than acting as a singular determinant of follicular fate, NLRP3 signaling may interact with endocrine, metabolic, and redox networks to influence developmental trajectories. The proposed transition framework provides a conceptual approach for understanding how disturbances in redox, metabolic, and inflammatory homeostasis may influence follicular developmental trajectories under pathological conditions. It should not be interpreted as a universal mechanism explaining all ovarian diseases. Future studies employing conditional genetic manipulation, temporal intervention, and multi-omics approaches will be essential to determine whether NLRP3 functions as a causal regulator, a permissive factor, or a downstream consequence of follicular stress.

## 9 Discussion and conclusion

Although accumulating evidence has established that the NLRP3 inflammasome plays an important role in oxidative stress, metabolic regulation, inflammation, and ovarian physiology, the concept proposed in this review that NLRP3 may function as a redox-metabolic integrator or gatekeeper regulating follicular fate determination, should currently be regarded as a hypothesis-driven conceptual framework rather than a fully validated biological model. While numerous experimental studies have demonstrated that NLRP3 activation is associated with granulosa cell pyroptosis, follicular atresia, ovulatory inflammation, ovarian aging, and reproductive disorders such as PCOS and POI, direct evidence demonstrating that NLRP3 causally determines follicular developmental decisions across multiple developmental stages remains limited.

An important conceptual distinction of this framework is that developmental transitions and inflammatory activation states represent separate analytical dimensions. Follicular growth, dominance acquisition, and ovulatory competence describe biological processes, whereas NLRP3 activation states describe cellular signaling conditions. Although these dimensions may interact, current evidence does not support the interpretation that NLRP3 activation levels directly define follicular developmental stages. Future studies should therefore evaluate the temporal and causal relationship between NLRP3 activity and specific follicular transitions.

Another important limitation concerns the dynamic nature of follicular development. Follicular fate is determined through the coordinated integration of endocrine hormones, cellular metabolism, mitochondrial function, immune responses, extracellular matrix remodeling, angiogenesis, and intercellular communication. It is therefore unlikely that NLRP3 functions as an isolated regulator. Instead, NLRP3 probably represents one critical signaling node embedded within a broader regulatory network that integrates redox balance, metabolic adaptation, hypoxic signaling, and inflammatory responses. Consequently, the proposed model should be interpreted as an integrative systems-level framework that facilitates understanding of how diverse microenvironmental cues may converge to influence follicular developmental trajectories, rather than implying that NLRP3 alone dictates follicular destiny.

Although HIF-1α, mTOR, NRF2, AMPK/SIRT1, and NLRP3 are frequently interconnected during cellular stress responses, the precise directionality and hierarchy among these pathways in follicular development remain incompletely defined. Current evidence supports reciprocal interactions rather than a simple linear cascade. Future studies employing temporal multi-omics approaches and pathway-specific perturbation will be necessary to establish causal relationships.

The proposed three-transition framework is intended to generate experimentally testable hypotheses rather than define fixed biological thresholds. Each transition represents a coordinated state characterized by multiple molecular, cellular, and physiological parameters. Importantly, the framework can be falsified if stage-specific manipulation of NLRP3 signaling fails to alter predicted developmental transitions or if alternative regulatory pathways fully compensate for NLRP3 disruption. Future studies integrating conditional genetic models, temporal perturbation, and quantitative systems biology approaches will be essential to determine whether NLRP3 represents a causal contributor, a permissive factor, or a consequence of follicular developmental changes (Table 1).

**TABLE 1:** Proposed experimental strategies to evaluate the hypothesis-driven NLRP3-associated follicular fate framework.

Biological transition	Conceptual hypothesis	Candidate measurable parameters	Experimental approaches	Expected Interpretation	Evidence status
Primordial follicle activation/Growth transition	Redox-metabolic signals and inflammatory adaptation may contribute to the initiation of follicular growth	FOXO3a phosphorylation, PI3K-AKT activation, granulosa proliferation, mitochondrial activity, ATP production	Oocyte/granulosa cell-specific NLRP3 knockout; inducible activation/inhibition; single-cell transcriptomics	Determine whether NLRP3 signaling is required or merely associated with follicle activation	Hypothesis; direct evidence lacking
Growing follicle maintenance	Physiological NLRP3 activity may participate in maintaining controlled inflammatory adaptation and cellular stress responses	NLRP3 expression, ASC assembly, IL-1β/IL-18 secretion, ROS levels, mitochondrial membrane potential	Primary granulosa cell culture; conditional genetic models; metabolic imaging	Define whether basal NLRP3 activity supports follicular competence	Emerging evidence
Dominant follicle selection	NLRP3-related inflammatory and metabolic signals may contribute to follicular competitiveness within a cohort	Gonadotropin responsiveness, FSH receptor signaling, steroidogenic genes, granulosa survival markers	Temporal analysis of follicular cohorts; spatial transcriptomics; hormone stimulation models	Test whether NLRP3 influences dominance acquisition or reflects downstream adaptation	Currently speculative
Ovulatory competence	NLRP3-associated inflammatory signaling may participate in LH-induced follicular remodeling	IL-1β, IL-18, MMP2/MMP9, ECM remodeling markers, cumulus expansion	LH-triggered ovulation models; NLRP3 inhibition or knockout; rescue experiments	Determine whether NLRP3 contributes to ovulatory remodeling	Partial evidence
Atresia transition	Excessive NLRP3 activation may promote inflammatory damage and granulosa cell dysfunction	Caspase-1 activation, GSDMD cleavage, apoptosis markers, ROS accumulation	Disease models (PCOS, toxicant exposure, aging); NLRP3 inhibition studies	Establish causal contribution to follicular degeneration	Supported by associative and pharmacological evidence
PCOS-associated dysfunction	Metabolic stress-induced NLRP3 activation may contribute to ovarian inflammatory imbalance	Insulin resistance markers, androgen levels, NLRP3/IL-1β signaling	PCOS animal models; granulosa cell models; intervention studies	Evaluate whether NLRP3 modulation improves ovarian phenotype	Cellular/animal evidence
POI-associated dysfunction	Oxidative stress and mitochondrial injury may activate NLRP3-related inflammatory responses	Follicular fluid cytokines, mitochondrial damage markers, ovarian reserve markers	Human cohort studies; POI models	Determine association between NLRP3 activity and ovarian decline	Limited clinical evidence
Ovarian aging	Chronic inflammaging may involve sustained NLRP3 activation and impaired mitochondrial homeostasis	mtDNA damage, ROS, senescence markers, follicle number	Aging mouse models; NLRP3 inhibition; longevity interventions	Test contribution of NLRP3 signaling to age-related ovarian dysfunction	Mainly animal evidence

The proposed framework does not assume that NLRP3 directly controls follicular fate. Instead, the listed experiments are designed to determine whether NLRP3 activity represents a causal regulator, a permissive factor, or a downstream consequence of follicular stress during different developmental stages.

Although increasing evidence supports an association between NLRP3-related inflammatory signaling and ovarian dysfunction, direct mechanistic evidence remains incomplete. Most available studies have not employed cell-type-specific knockout models, stage-specific manipulation, or rescue strategies capable of distinguishing causal regulation from secondary activation. Therefore, the proposed framework should be interpreted as a hypothesis-generating model. Future studies combining conditional genetic approaches, temporal perturbation, and multi-dimensional molecular profiling will be necessary to determine the precise role of NLRP3 during different stages of follicular development.

From a translational perspective, future studies should determine whether the proposed model can improve disease classification and therapeutic stratification in ovarian disorders. Rather than simply evaluating NLRP3 inhibition as an anti-inflammatory strategy (Berkel, 2024; Kavarthapu et al., 2026; Zhou et al., 2024; Grzeczka et al., 2025; Yang et al., 2024; Wu et al., 2022; Zhu et al., 2023; Murakami et al., 2022), future clinical investigations may explore personalized interventions targeting the entire redox-metabolic-inflammatory regulatory network, including antioxidant therapy, mitochondrial protection, metabolic reprogramming, inflammasome modulation, and endocrine optimization.

Ultimately, rigorous experimental validation of this proposed framework may establish a systems-level understanding of follicular fate regulation and provide a theoretical foundation for precision medicine in female reproductive disorders.
